# The Low Toxicity of Graphene Quantum Dots is Reflected by Marginal Gene Expression Changes of Primary Human Hematopoietic Stem Cells

**DOI:** 10.1038/s41598-019-48567-6

**Published:** 2019-08-19

**Authors:** Stefan Fasbender, Lisa Zimmermann, Ron-Patrick Cadeddu, Martina Luysberg, Bastian Moll, Christoph Janiak, Thomas Heinzel, Rainer Haas

**Affiliations:** 1 Condensed Matter Physics Laboratory, Heinrich-Heine-University, D-40204 Düsseldorf, Germany; 2 Department of Haematology, Oncology and Clinical Immunology, Heinrich-Heine-University, D-40204 Düsseldorf, Germany; 3 Ernst Ruska Centre, Jülich Research Centre, D-52425 Jülich, Germany; 4 Institute for Inorganic Chemistry and Structural Chemistry, Heinrich-Heine-University, D-40204 Düsseldorf, Germany

**Keywords:** Biomaterials, Translational research, Nanoparticles

## Abstract

Graphene quantum dots (GQDs) are a promising next generation nanomaterial with manifold biomedical applications. For real world applications, comprehensive studies on their influence on the functionality of primary human cells are mandatory. Here, we report the effects of GQDs on the transcriptome of CD34^+^ hematopoietic stem cells after an incubation time of 36 hours. Of the 20 800 recorded gene expressions, only one, namely the selenoprotein W, 1, is changed by the GQDs in direct comparison to CD34^+^ hematopoietic stem cells cultivated without GQDs. Only a meta analysis reveals that the expression of 1171 genes is weakly affected, taking into account the more prominent changes just by the cell culture. Eight corresponding, weakly affected signaling pathways are identified, which include, but are not limited to, the triggering of apoptosis. These results suggest that GQDs with sizes in the range of a few nanometers hardly influence the CD34^+^ cells on the transcriptome level after 36 h of incubation, thereby demonstrating their high usability for *in vivo* studies, such as fluorescence labeling or delivery protocols, without strong effects on the functional status of the cells.

## Introduction

A wide variety of nanomaterials has been introduced into our daily life to improve the properties of consumer products like clothes^1^, food^2^ and cosmetics^3^. Despite a large body of studies, their potential influence on health is often poorly understood^4,5^. After its discovery in 2004, graphene has gained much attention as a novel two dimensional nanomaterial^6^. Due to its superior mechanical, chemical and electronic properties^7^, many graphene containing consumer products have emerged in the packaging and sports industry^8,9^.

Graphene quantum dots (GQDs) are a nanometer-sized derivative of one or a few layers of graphene. Because of their large surface to mass ratio, they are a fluorescent nanomaterial with a broad spectrum of applications in the field of organic photovoltaic devices, catalysis, sensors and biomedicine^10^. In particular, the field of biomedicine offers many opportunities as GQDs enter the cytoplasm not only of human cell lines, but also of primary human blood cells, without significant effects on cell viability^11–15^. Therefore, GQDs have been used in research related to long term and deep tissue imaging, cancer diagnostics, intracellular sensing and drug delivery^16–21^. As far as the exposure of human cells *in vitro* or *in vivo* to GQDs is concerned, their possible side effects on the functionality of these cells remain a question of ongoing research. For instance, *in vivo* studies show that high doses of GQDs disrupt the progression of embryonic development in zebrafish^22^. In mice, intravenously injected larger graphene nanosheets induced Th2 inflammatory responses^23^. *In vitro* studies using fibroblast cell lines show increased expression of p53, Rad51 and OGG1 proteins, indicating DNA damage caused by GQDs of 40 nm diameter^24^. The toxicity of graphene based nanomaterials thus appears to be mainly related to particle size, surface functional groups, oxygen content, surface charges and impurities, while the formation of reactive oxygen species (ROS) seems to be the most common underlying mechanism^25^. However, these toxicity studies reported hitherto are no more than a glimpse of the overall, possibly quite complex, responses of the cells to the incubation with GQDs. In particular, nothing is known about the underlying signaling pathways. As has been pointed out recently, a more comprehensive picture requires a gene expression analysis, carried out on primary human cells instead of cell lines^26^.

In the study presented here, we report the cellular gene expression and the attributed signaling pathways after incubation of primary human CD34^+^ hematopoietic stem cells (HSCs) with a high concentration (500 μg ml^−1^) of GQDs for an exposure time of 36 hours. HSCs are particularly susceptible to any kind of cytotoxic effects such as conventional chemotherapy or radiation. They are composed of the most primitive hematopoietic stem cells as well as the more committed progenitor subset, which is responsible for the lifelong production of mature blood cells.

## Results and Discussion

### Preparation and characterization of the graphene quantum dots

**Figure 1:**
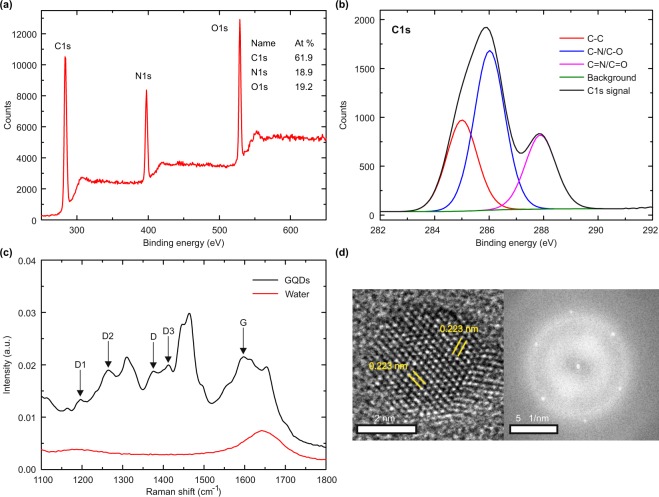
(**a**) Survey XPS spectrum of the GQDs quantifying the amount of C (61.9%), N (18.9%) and O (19.2%). (**b**) C1s XPS spectrum of the GQDs revealing C-C, C-O and C-N bonds. (**c**) Raman spectrum of the GQDs dispersed in water (black trace) and of water (red trace). (**d**) HRTEM image (left) of one GQD and the corresponding Fast Fourier Transform (right).

The GQDs were prepared by pyrolysis of citric acid, following a modified version of the well-established recipe of Qu *et al*.^27^ using a well-defined microwave assisted hydrothermal synthesis process. Before their application to the cells, the GQDs underwent a targeted characterization to determine their chemical composition and size as well as their fluorescence properties. They consist of 40% C, 19% N and 8% H (mass fraction) as determined by CHN chemical elemental analysis. The remaining fraction can be assigned to O, as the GQDs consist of no other elements. This was confirmed by X-ray photoelectron spectroscopy (XPS) (Fig. 1(a)). Further analysis of the C1s resonance (see Fig. 1(b)) indicates that C-C bonds amount to 29% of the carbon bonds, while the remaining bonds are approximately equally distributed among C-O and C-N bonds, which is confirmed by the N1s and O1s spectra (Fig. S1). The XPS analysis thus proves that nitrogen compounds, an essential ingredient for the anticipated fluorescence at long wavelengths, have been incorporated into the GQDs.

The Raman spectra of the water-dispersed GQDs (Fig. 1(c)) show some functional groups from citric acid and diethylentriamine (DETA), which is in good agreement with the XPS analysis. Raman signals at 1657, 1411, 1053, 943 and 785 cm^−1^ match with vibrations of citric acid. Signals at 1463, 1309 and 1091 cm^−1^ match with diethylenetriamine vibrations (see Fig. S2). We can also assign signals at 2946 and 2976 cm^−1^ to vibrations of citric acid and DETA. Two bands are visible at 1375 and 1596 cm^−1^ which we assign to the D and G band signals known for graphene quantum dots^28^. Furthermore, there are peaks at 1195, 1264 and 1412 cm^−1^ labeled D1, D2 and D3, which can be assigned to sp2-sp3 carbon, COOH/C-OH and C=O/C-O edge functional groups, respectively^29^. This indicates that the C-C part of the C1s XPS peak is composed of both sp2 and sp3 hybridized carbon. A detailed comparison of the Raman spectra of GQDs, DETA, citric acid and water is shown in Fig. S2.

For high resolution transmission electron microscopy (HRTEM) the GQDs were dispersed on an ultrathin amorphous graphite substrate. On a larger scale (see Fig. S3(a)), the GQDs appear as randomly distributed, dark spots. The size histogram indicates an average diameter of 3.3 nm with a full width at half maximum of 0.6 nm (see inset of Fig. S3(a)). Atomic resolution was observed only for a few GQDs, which had an appropriate orientation with respect to the substrate plane. A typical result is shown in the left part of Fig. 1(d), where a hexagonal structure with a lattice constant of 0.223 nm was found, which is 10% smaller than the lattice constant of graphene (0.246 nm). More generally, we observed particles with hexagonal symmetry and lattice constants in the range between 0.21 nm and 0.25 nm, in good agreement with the values published earlier^27,30^. This is also in line with the observation that the lattice constant of graphene decreases as other types of atoms are attached^31^. The fuzzy edge may indicate the presence of defects and/or functional groups, in qualitative agreement with the XPS analysis. The Fast Fourier transform (FFT) in the right part of Fig. 1(d) confirms a crystalline structure with a six-fold symmetry, while the X-ray diffraction (XRD) pattern of the GQDs (Fig. S3(b)) shows a broad peak around 22° that is usually observed for GQDs^28,32,33^ and two distinct peaks at 21.9° and 26.2° that can be assigned to elemental carbon and graphite^34^. We also performed topographical measurements with an atomic force microscope (AFM) (see Fig. S4). Particle heights between 1 nm and 2 nm are measured, which is in agreement with results reported earlier for two and three layers of graphene. Based on these findings, we conclude that our GQDs have a disk-like, flat shape, consist mostly of carbon, with equal contents of oxygen and nitrogen and have a predominantly hexagonal crystal structure.

**Figure 2:**
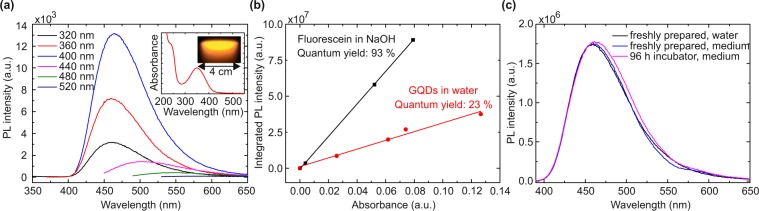
(**a**) Fluorescence spectra of the quantum dots as a function of the excitation wavelength. The corresponding absorbance spectrum including an image of the GQD solution under excitation with blue light is shown in the inset. (**b**) Integrated PL intensity vs. absorbance of fluorescein and GQDs to determine the GQD quantum yield of 23% at 360 nm excitation. (**c**) Comparison of the fluorescence spectrum of freshly prepared GQDs and GQDs stored for 96 h in the incubator in a humidified atmosphere at 5% CO_2_ and 37 °C dissolved in cell culture medium at 360 nm excitation.

The relevant fluorescence properties of the GQDs are summarized in Fig. 2. As the excitation wavelength is increased from 320 nm to 400 nm, the shape and maximum of the emission spectrum (at a wavelength of 460 nm) remains almost unchanged, while the emission intensity increases by a factor of approximately 4. A further increase of the excitation wavelength causes an abrupt change of the emission spectrum to larger emission wavelengths, accompanied by a marked decrease of the fluorescence intensity. For example, at an excitation wavelength of 480 nm, the maximum of the emission spectrum occurs at 560 nm, thereby showing that the fluorescence mechanisms depend on the excitation wavelength. Qu *et al*.^27^ have attributed the excitation that leads to the fluorescence in the blue and belongs to the absorbance peak around 350 nm (inset) to the n-π* transition of C=O, whereas that one that causes the fluorescence in the yellow was attributed to the n-π* transition of the N state. In the experiments reported below, we used the emission at larger wavelength, thereby avoiding irradiation of the cells by UV light and remaining compliant with most medical devices using fluorescence detection. The quantum yield of the GQDs at the absorbance maximum of 360 nm was determined to be 23% (Fig. 2(b)). Furthermore, neither the fluorescence spectrum of the GQDs nor its intensity changed significantly over a time period of 96 h when they were dissolved in cell culture medium and stored in the incubator in a humidified atmosphere at 5% CO_2_ and 37 °C (Fig. 2(c)).

### Cultivation of CD34^+^ cells and GQD uptake

**Figure 3:**
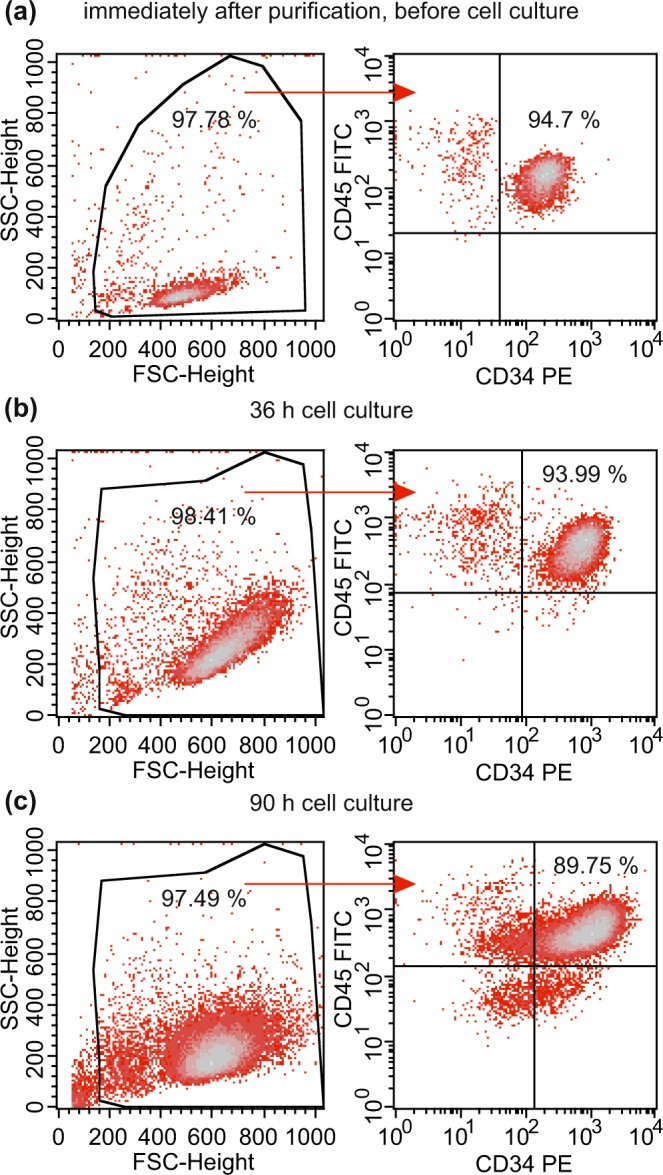
Fraction of CD34^+^ following selection using anti-CD34 MoAb coated immunomagnetic beads at time point 0 (**a**), after 36 h (**b**) and after 90 h of cell culture (**c**). Viable cells were gated using a forward scatter (FSC) vs side scatter (SSC) plot. The fraction of CD34^+^ cells was obtained in a CD34 vs CD45 plot based on all viable cells.

Using the magnetic cell separation technology for the enrichment of CD34^+^ cells from leukapheresis products of healthy donors, we obtained populations with a purity of at least 90% as measured by flow cytometry (Fig. 3(a)). For the cultivation of the stem and progenitor cells, we used Stem SPAN™ SFEM II medium combined with Stem SPAN™ CD34^+^ Expansion Supplement, a specific medium favoring self-renewal rather than differentiation. As a result, the decrease in the proportion of CD34^+^ cells following 36 h of cultivation was marginal with a significant reduction only after 90 h, see Fig. 3(b,c). A broadening of the population regarding size and granularity, see the FSC and SSC data in Fig. 3(c), reflects the distinct alteration of the cells as well. The time-dependent changes observed within the CD34^+^ cell populations were identical to those when the cells were cultivated in the presence of GQDs at a concentration of 500 μg ml^−1^ as shown in Fig. S5.

**Figure 4:**
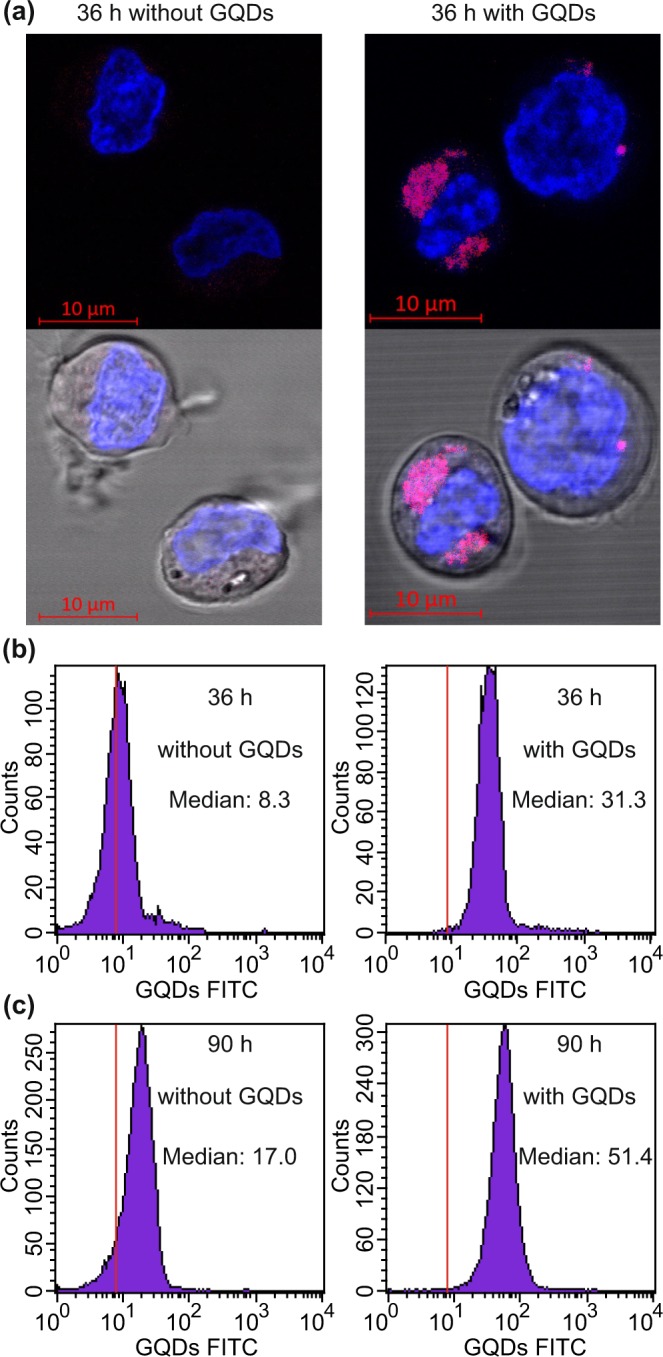
(**a**) Confocal fluorescence microscopy images of living cells cultured for 36 h without GQDs (left) and with GQDs (right) as observed (top row) and merged with the bright field images (bottom row). The stained nuclei are represented in blue while GQDs appear in purple. The fluorescence intensity distributions as determined by flow cytometry after 36 and 90 hours of cultivation are shown in (**b**,**c**) respectively. Here, the left parts show the evolution just in the culture medium and the right parts that one in the presence of the GQDs.

To visualize the uptake of the GQDs into the cells, we performed confocal fluorescence microscopy on living CD34^+^ cells, that were stained with Hoechst 33342 for representation of the nuclei. A representative example is given in Fig. 4(a), where the fluorescence images of the cells and the merged bright field images, representing the Hoechst 33342 channel (405 nm excitation, 410–495 nm emission) in blue and the GQD channel (488 nm excitation, 495 nm–630 nm emission) in red, are depicted. The GQDs appear purple as they also emit in the Hoechst 33342 channel. Distinct areas of purple color were observed only in the population cultured in the presence of GQDs. The spatial distribution of the fluorescence intensity implies that the GQDs accumulate in regions near the nucleus. Earlier work suggests that the GQDs are confined to lysosomes^35^. Also, an accumulation of the GQD fluorescence at the Golgi apparatus has been reported recently^36,37^. As a result of the cellular uptake, a time dependent shift of the fluorescence intensity emerging from the cells, as measured by flow cytometry, was observed, with an increase of the median from 8.3 (36 h without GQDs) to 31.3 (36 h with GQDs) and a maximum value of 51.4 following 90 h of incubation with GQDs (Fig. 4(b,c)). Notably, the autofluorescence intensity of the cells cultured without GQDs increased by more than a factor of two during that time, namely from a median value of 8.3 to 17.0. Taking into account the observed changes of the CD34^+^ cell population and the decrease of the CD34^+^ proportion after 90 h, we opted for a cultivation time of 36 h for the gene expression analysis.

### Gene expression analysis

**Figure 5:**
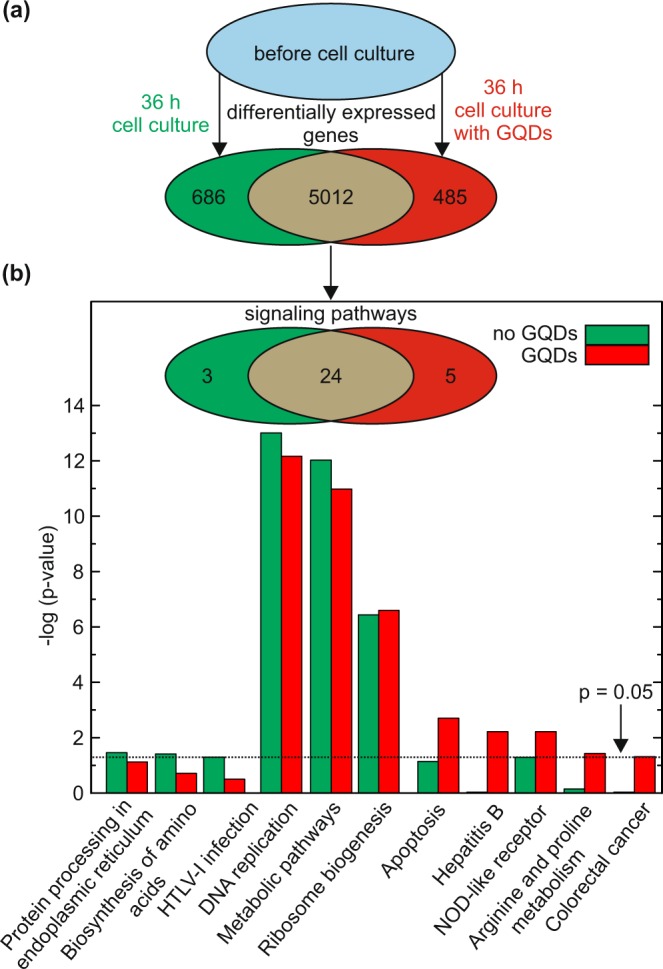
Sketch of the design and the result of the gene expression experiment. The transcriptome of the CD34^+^ cells was measured before and after exposure of the cells to the two experimental conditions. The modified gene expressions are sorted according to the ratio of their amplification in the presence vs. the absence of the GQDs in the culture. The expression of 5012 genes was changed under both culture conditions, while the expression of 485 genes was only changed in the presence of GQDs and the expression of 686 genes only differed when the CD34^+^ cells were cultivated without GQDs. (**b**) Allocation of the changes to 24, 5 and 3 affected signaling pathways, respectively (inset) and the -log p - values of the 8 pathways with significant response to the GQDs (main figure). Also shown is the effect on the pathways DNA replication, metabolic and ribosome biogenesis.

In order to assess the effect of GQDs on the transcriptome, we isolated the CD34^+^ cells of four healthy donors and took one third of the freshly isolated CD34^+^ cells aside to extract the RNA using the RNeasy Mini Kit. The remaining cells were cultivated for 36 hours with and without GQDs at a concentration of 500 μg ml^−1^. Independent of the presence of GQDs, we observed a twofold mean increase in the concentration of CD34^+^ cells, demonstrating that the cells underwent one cell cycle on average during the cultivation interval. Afterwards, the RNA of the CD34^+^ cells, cultivated with and without GQDs, was extracted. Using capillary electrophoresis, the RNA integrity of all 12 samples was determined with an average RIN (RNA integrity number) of 9.7, whereby a value of 10.0 implies the highest integrity (see Table S1 for the RIN numbers of all samples). Finally, the RNA was processed for transcriptome-wide gene-level expression profiling on the Clariom™ S micro array. Using the Transcriptome Analysis Console (TAC) 4.0 software and iPathway guide, we conducted a meta-analysis and compared the measurement results obtained after the CD34^+^ cell cultivation under both conditions, with those observed in CD34^+^ cells before the cells were put into the culture medium, see Fig. 5(a). This kind of comparison strikes us as mandatory, since a comparison restricted to the two culture conditions directly after the 36 hour cultivation may obscure subtle effects of the GQDs, namely in case of predominant changes related to the cultivation per se. The thresholds for differential expression were (i) an absolute value of the fold change >1.5, and (ii) a false discovery rate (FDR) - adjusted p-value < 0.05, using the Benjamini - Hochberg method^38^ to correct for multiple comparisons.

We are not aware of studies regarding the effect of the cell culture on the gene expression of primary human stem cells. Such an effect, however, can be expected since the cells are deprived of their natural environment. Remarkably, the cultivation per se leads to significant changes in the gene expression pattern of 5012 genes, corresponding to 24% of the genes contained within the array. Although the expression of these genes within the CD34^+^ cell population has significantly changed following the 36 hour of cultivation under both conditions, the changes induced may differ with regard to their sign as well as their amplitude. Using iPathway guide for a bioinformatic analysis, the changes observed within the transcriptome could be allocated to 24 out of the 320 assignable signaling pathways. For this assessment, we applied the Bonferroni correction to adjust for multiple comparisons. Among the most prominent pathways were those associated with DNA replication and cell metabolism. Albeit interesting in themselves, these effects are not at the focus of the present work and will not be considered in more detail here.

We proceed with the overall assessment of the transcriptome. 485 genes were identified that show significant changes exclusively in the GQD - containing cultures. On the other hand, 686 differentially expressed genes were observed only under culture conditions without GQDs, i.e., these expressions are suppressed by the presence of the GQDs. This corresponds to eight modified signaling pathways. Five of them, namely (1) Apoptosis, (2) Hepatitis B, (3) NOD-like receptor signaling, (4) Arginine and proline metabolism and (5) colorectal cancer, showed a change in the presence of the GQDs, while in the remaining three, (6) Protein processing in endoplasmic reticulum, (7) Biosynthesis of amino acids and (8) HTLV-I infection, the changes were suppressed by the GQDs, see Fig. 5(b).

The mere number of affected pathways does not reflect the strength of the GQD related effects. In order to quantify the impact of the GQDs, we determined the negative logarithms of the Bonferroni corrected p-values^39^. We limit our discussion to the eight pathways affected only in one of the two culture conditions, plus the three most prominent pathways affected in both culture conditions. The full set of the 32 pathways is provided in Table S2.

**Figure 6:**
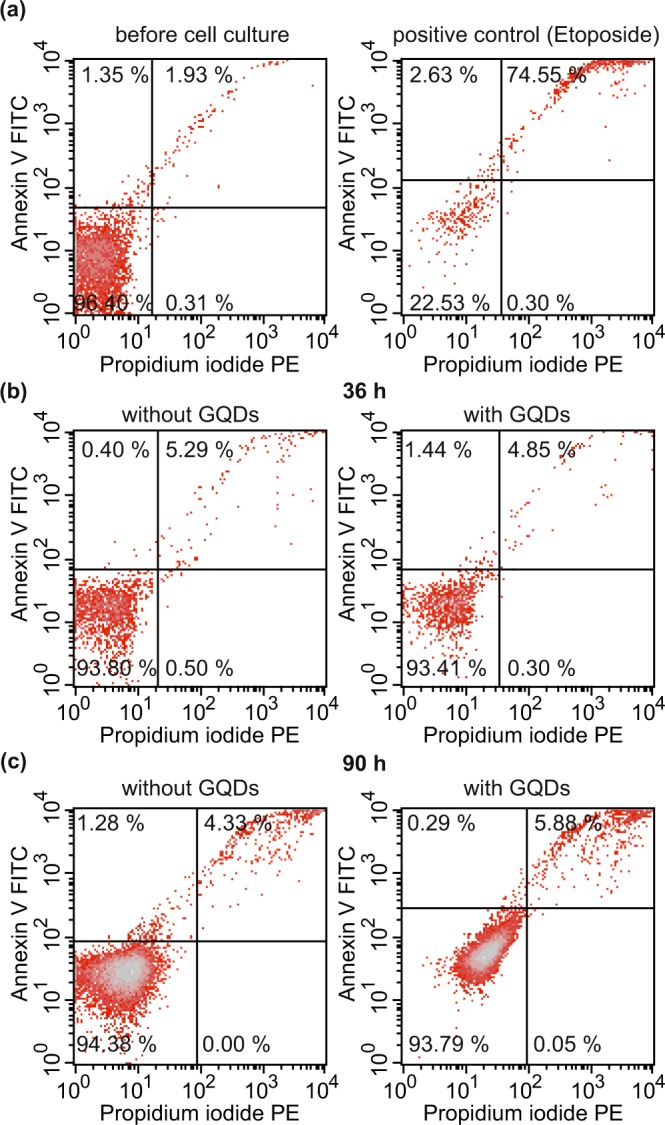
Annexin apoptosis assay for (**a**) untreated CD34^+^ cells before cell culture (left) and a positive control using Etoposide as an apoptosis inducing agent (right), (**b**) CD34^+^ cells cultivated for 36 h without (left) and with GQDs (right) and (**c**) CD34^+^ cells cultivated for 90 h without (left) and with GQDs (right). Shown is one representative example out of 3 independent experiments.

In general, the attribution of the gene expression to the signaling pathways is only qualitative and does not necessarily imply a change of the amplitude of the corresponding cellular processes. Therefore, we selected one of the pathways for an independent, quantitative verification, namely the apoptosis pathway, since it is not only a good measure for cell damage, but also can be tested via the well-established Annexin V/Propidium Iodide assay. As shown in Fig. 6, this pathway got in fact activated in both culture conditions, even though the effect was very small (less than 5%). However, there was no significant difference between the proportions of Annexin expressing CD34^+^ cells when the cells were cultivated for 36 h (Fig. 6(b)) in the presence of GQDs compared to those without them. Since it may take longer for the changed gene expression to get translated into apoptosis, we also carried out the assay after 90 h, see Fig. 6(c). Also here, no significant difference between the two culture conditions could be detected. Thus, the significance in the apoptosis pathway is confirmed by the Annexin array, but the p-values difference between the two culture conditions cannot be seen in the direct test.

We proceed by describing the identification of the genes with the highest susceptibility to GQDs and the direct comparison of the CD34^+^ cell samples from the two culture conditions. We find 33 genes that meet the criteria required for being regarded as differentially expressed before the FDR correction, namely an absolute value of the fold change larger than 1.5, and a p-value below 0.05, see Table S3 for a comprehensive set of data. 32 of these genes showed only marginal changes with a fold change <2. After the correction using the FDR algorithm, just one gene, namely Selenoprotein W, 1 (SEPW1), showed a significant differential expression according to the criterion given above (|fold change| > 1.5 and FDR < 0.05).

**Figure 7:**
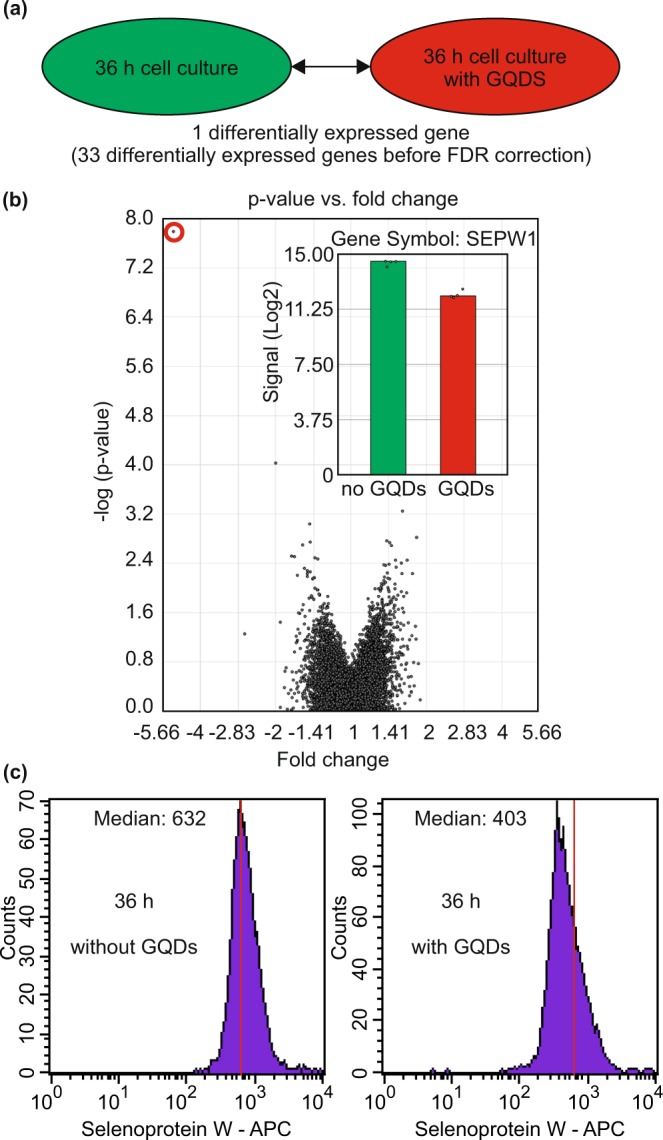
(**a**) Comparison of the transcriptome of CD34^+^ cells following cultivation under the two conditions for 36 hours. Only the SEPW1 gene showed a significant differential expression. (**b**) Volcano plot for all genes (SEPW1 is labeled with a red circle in the upper left corner) and sample signals for the SEPW1 gene (inset). (**c**) Measurement of the intracellular amount of Selenoprotein W following 36 h of cultivation without GQDs (left) and with GQDs (right).

The SEPW1 gene shows a 5-fold smaller expression level following cultivation in the presence of GQDs as compared to the cultivation in the absence of GQDs. The comparative impact on the expression level of this single gene can be seen in the volcano plot (Fig. 7(a,b)). Since the SEPW1 gene encodes directly the production of the Selenoprotein W, the intracellular amount of this protein after 36 h of cell culture was measured by flow cytometry, using an AF647 conjugated Anti-Selenoprotein W antibody, see Fig. 7(c). A marked decrease of the median fluorescence intensity in the APC channel (635 nm excitation, 660 ± 20 nm emission) from 632 (without GQDs) to 403 (with GQDs) was observed, thereby verifying the results from the gene expression analysis.

We continue by interpreting these observations in the context of stem cell biology. At the beginning of the incubation experiment, the majority of the CD34^+^ cells are in the G1 phase of the cell cycle, while in the bone marrow, significantly more CD34^+^/Ki-67^+^ cells are in the S/G2M phase^40^. Being transferred into a liquid culture medium and thereby deprived of their natural microenvironment within the BM niche, they start to proliferate. This is in line with our observation that following a cultivation period of 36 hours, the cell number doubles approximately, irrespective whether GQDs were present in the medium or not. The expression pattern resembles to some extent that one of CD34^+^ cells residing in the bone marrow, in comparison to those circulating in the peripheral blood. For instance, greater cell cycle and DNA synthesis activity of BM-CD34^+^ than PB-CD34^+^ cells were reflected by the 2- to 5-fold higher expression of nine genes involved in cell cycle progression, eleven genes regulating DNA synthesis and the cell cycle-initiating transcription factor E2F-1^41^.

Among the 5012 common cultivation-related genes, only SEPW1 was significantly modified by the presence of the GQDs, with a strongly reduced expression level. The function of this gene has been shown to be related to the control of cell cycle entry. Hawkes *et al*. identified targets of selenium (Se) in cultured human breast and prostate epithelial cells using gene expression by DNA microarrays^42^. SEPW1 was the only selenoprotein messenger RNA (mRNA) increased by both sodium selenite (specific) and high-Se serum (physiologic). Interestingly, SEPW1 small interfering RNA inhibited G1-phase progression and increased G1-phase gene transcripts, while decreasing S-phase and G2/M-phase gene transcripts. These observations imply cell cycle interruption at the G1/S transition. Along this line of reasoning, our finding could indicate that the cultivation-induced upregulation of SEPW1 is counteracted - similar to the effect of small interfering RNA - by the GQDs with an inhibitory effect on cell cycle progression, i.e., cell division. Still, the effect was not sufficiently strong enough to be translated into a functional effect, as there was no difference in the concentration of CD34^+^ cells cultivated in the presence of GQDs or alone. Regarding apoptosis, one report shows that large graphitic flakes with diameters in the range of 300 to 1 cause apoptosis in red blood cells and in macrophages, most likely through generation of intracellular reactive oxygen species (ROS)^43^, which is in line with earlier studies on skin fibroblasts^44^. GQDs also induced apoptosis and inflammatory reactions in macrophages^45^. In contrast to these reports, we did not detect an increased, GQD - induced apoptosis in the primary human HSCs. Furthermore, none of the other seven modified pathways is readily associated with cellular damage or repair, which speaks against toxicity beyond apoptosis. Interestingly, in some studies the low toxicity appears to be independent of the attached functional groups, as carbon nanodots with various amounts of nitrogen and oxygen do not affect the cell viability in a significant way^28,46^, while in other studies a distinct toxicity of some functional groups is observed^47^.

This small effect of incubated GQDs on the transcriptome of the cells, including triggering of apoptosis, is striking and asks for an explanation. We tentatively interpret this relative inertness by referring to recent reports on the subcellular distribution of GQDs inside cells after incubation. It has emerged that the GQDs are encapsulated in vesicles like lysosomes^35^, which is in tune with the observed accumulation in the periphery of the nucleus (Fig. 3(a)). Thus, it is conceivable that encapsulation not only protects the compartments of a cell from the possible effects of the GQDs but also renders the attached functional units as not very relevant, provided they do not modify the compartementalization. It should be emphasized that in other publications that report an approximately homogeneous distribution of the GQDs across the cytoplasm, the cells have been fixed, i.e., they are not alive at the time of the imaging. Our confocal fluorescence images rather imply that in living cells, the GQDs accumulate in the periphery of the nucleus, in tune with recent observations^36,37^. Larger GQDs, on the other hand, may be encapsulated to a lesser degree or even damage the cell membrane during incubation. Nevertheless, even though the GQDs appear to be well separated from the cell compartments, GQD - induced drug delivery is achievable, namely regarding applications directly in endosomes or lysosomes, as well as schemes where the drug is released from lysosomes into the nucleus^35^.

The functional relevance of the other differentially expressed genes within the pathways of Arginine and Proline, Colon Cancer, Hepatitis B pathway, protein processing in the endoplasmic reticulum, biosynthesis of amino acids, HTLV I infection and NOD like receptor is, based on the knowledge acquired here, hard to address adequately as they encompass a broad variety of overlapping genes involved in very general cellular processes such as K-RAS, JNK and c-Jun. Such an interpretation would require further, complementary experiments.

## Summary and Conclusions

We examined the *in vitro* effects of small graphene quantum dots (with diameters of approximately 3 nm) on the gene expression of primary human cells, namely blood-derived CD34^+^ cells from leukapheresis products of normal donors, after incubation. Surprisingly, of the 20800 genes included in our study, only a single gene is strongly affected, i.e., SEPW1 is downregulated with a fold change of −5. Even though this downregulation might be related to a slowdown of the cell cycle, this was not reflected in a decreased proliferation. Furthermore, 1170 gene expressions are weakly affected and ascribed to eight signaling pathways. The effect of the GQDs on the transcriptome is markedly weaker than that one of the culture medium, which affects 5012 gene expressions belonging to 24 signaling pathways.

We conclude that our GQDs show only marginal effects on the transcriptome as well as low toxicity. The cells used form a rare hematopoietic stem cell population that is usually residing in the bone marrow, are highly sensitive to environmental disturbances and they may therefore be regarded as a particular sensitive type of cell for studying the effects of GQD exposure. Furthermore, we excluded influences on the results by alterations of the cellular phenotype, since we have concentrated our study on the early stage of cultivation (36 h).

These results are commensurate with recent observations that after incubation, the GQDs are encapsulated by vesicles inside the cell, possibly in relation to the endosomal - lysosomal evolution after uptake of extracellular particles via endocytosis. Possibly, such a compartmentalization protects the cell from possibly toxic effects of the GQDs, irrespective of the functional groups they carry. This situation may be useful for some diagnostic or therapeutic applications, while for others, endosomal release, and the corresponding toxicity studies thereafter, would be required.

## Materials and Methods

### Materials

Citric acid (ACS reagent, ≥99.5%), Diethylentriamine (DETA, 99%), L-Glutamine-Penicillin-Streptomycin solution, Dulbecco’s Phosphate Buffered Saline (DPBS), Float-A-Lyzer dialysis devices (100–500 Da), human serum albumin, EDTA, Selenoprotein-AF647 antibodies and sterile filters (200 nm) were obtained from VWR and antibodies against CD45-FITC/CD34-PE, CD34-APC and the FITC Annexin V Apoptosis Detection Kit I were purchased from BD biosciences. Stem SPAN™ SFEM II medium, Stem SPAN™ CD34^+^ Expansion Supplement (10x) and Lymphoprep™ solution were bought at STEMCELL™ Technologies and microwave reaction vessels were obtained from CEM GmbH. The CD34 MicroBead Kit UltraPure human, MACS LS columns and 30 pre separation filters were purchased from Miltenyi Biotec and the Fix and Perm Kit was bought from Thermo Fisher Scientific. Separation buffer was prepared freshly by supplementing 500 ml DPBS with 1.5 ml 5% human serum albumin and 1.5 ml 50 mM EDTA.

### Synthesis of graphene quantum dots (GQDs)

Fluorescent GQDs were synthesized using the recipe of Qu *et al*.^27^ with slight modifications. 210 mg citric acid and 340 mg DETA were placed into a 10 ml microwave reaction vessel and stirred for 10 min. The mixture was heated to 180 °C under constant stirring in the closed and pressure resistant vessel for 2 min using a CEM Discover Microwave Synthesizer. A viscous, dark orange liquid was obtained and dissolved with 10 ml DI water immediately after the cooldown. The aqueous solution was centrifuged with an Eppendorf MiniSpin^®^ at 13400 rpm for 10 minutes to remove insoluble residual. A Float-A-Lyzer dialysis device (MWCO 100–500 Da) was used to remove citric acid and DETA waste as well as the smallest particles by dialyzing 10 ml of the GQD solution against 2 l of DI water for 48 h with one water exchange after 24 h. The obtained pure GQD solution was dried and weighed with a Sartorius A 200S electronic analytical balance.

### Characterization of GQDs

Photoluminescence properties were measured using a Horiba FluoroMax^®^-4 spectrofluorometer, which allows for the correction of inhomogeneities in the instrument and detector response as well as differing lamp intensity, and absorbance spectra were taken with an Agilent Cary 4000 spectrophotometer. Thus, the fluorescence intensities are quantitavely comparable. AFM measurements were performed using the tapping mode of a Veeco 3100 with SiO_2_ as substrate. XPS data were measured using a PHI 5000 Versaprobe II XPS microprobe instrument with Au as substrate and TEM images were taken with the Titan G3 50-300 PICO^48^ on ultra thin amorphous carbon TEM grids. Raman spectra were obtained on a Bruker MultiRAM-FT Raman spectrometer equipped with a ND:YAG-laser (excitation wavelength 1064 nm). GQDs were measured in water dispersion in a mirrored cuvette with a laser power of 950 mW for 5000 scans with a resolution of 4 cm^−1^. Water reference spectra were obtained using the same settings. Solid-state spectra of citric acid were collected at 75 mW for 2500 scans with a resolution of 4 cm^−1^. The diethylenetriamine reference Raman spectrum was measured in an NMR tube at 400 mW for 2500 scans with a resolution of 4 cm^−1^. PXRD patterns were recorded on a Bruker AXS D2 Phaser using Cu-K*α*1/*α*2 radiation with *λ* = 1.5418 Å at 30 kV. Elemental analyses were conducted with a PerkinElmer CHN 2400 Analyzer.

### Collection of CD34^+^ primary human hematopoietic stem cells

Primary human hematopoietic stem cells were collected from leukapheresis products of in total seven healthy individuals who served as HLA-identical donors for an allogeneic blood stem cell transplantation using the common G-CSF conditioning at a dose of 480 μg per day over a period of 5 days. For isolation of mononuclear cells 1 ml of leukapheresis product was diluted with 5 ml DPBS and layered over 15 ml of Lymphoprep™ solution in a 50 ml conical tube for density gradient centrifugation. The tube was centrifuged at 835 g for 20 min at 20 °C without brake. The interphase was collected and layered again on 15 ml of Lymphoprep™ solution in a 50 ml conical tube followed by a second centrifugation at 835 g for 20 min at 20 °C without brake. To obtain a pellet of mononuclear cells the interphase was collected again and washed with 50 ml DPBS followed by 5 min centrifugation at 300 g. For the lysis of red blood cells the pellet was resuspended in 10 ml ammonium chloride solution (pH = 7.4). After 10 minutes the conical tube was stocked up to 50 ml with separation buffer and centrifuged for 5 min at 300 g. The obtained cell pellet was resuspended in 50 ml separation buffer and centrifuged again for 5 min at 300 g. To isolate the CD34^+^ hematopoietic stem cells from other mononuclear cells the pellet was resuspended in 300 μl separation buffer per 10^8^ cells and the obtained cell solution was incubated with 100 μl FcR blocking reagent and 100 μl CD34 MicroBeads UltraPure per 10^8^ cells respectively. After 30 minutes of incubation time at 4 °C the cells were washed with 50 ml of separation buffer twice (centrifugation for 5 min at 300 g) and resuspended in 3 ml separation buffer. The LS column with the pre-separation filter on top was placed in the magnetic field of a MidiMACS Separator and rinsed with 3 ml separation buffer, before the cell suspension was applied. Afterwards, unlabeled cells were removed by washing the column twice with 5 ml separation buffer and the column was placed on a 15 ml collection tube. Finally, the labeled cells were flushed out immediately with 7 ml separation buffer by pushing the plunger into the column.

### Cell cultivation of CD34^+^ primary human hematopoietic stem cells with GQDs

Using 24 well plates, CD34^+^ primary human hematopoietic stem cells were taken in culture immediately after collection. The culture medium contained 90% Stem SPAN™ SFEM II medium and 10% Stem SPAN™ CD34^+^ Expansion Supplement (10x) complemented by 1% L-glutamine-penicillin-streptomycin solution. Per well, 500 μl of cell suspension were dispensed at a concentration of 4 × 10^5^ cells/ml. For the culture with GQDs, we dissolved GQDs in culture medium at a concentration of 3 mg ml^−1^ and the obtained solution was sterile filtered with a 200 nm filter. 100 μl of the sterile GQD solution were added to all GQD culture wells (leading to a final GQD concentration of 500 μg ml^−1^) while the controls received 100 μl pure culture medium. The cultivation took place in a Heracell TM 150i incubator in a humidified atmosphere at 5% CO_2_ and 37 °C.

### RNA extraction

Total RNA was extracted using the Qiagen RNeasy Mini Kit. For lysis, the cells were washed twice with PBS and resuspended in 350 μl RLT buffer (including 1% *β*-mercaptoethanol). The lysate was given into a QIAshredder column (placed in a 2 ml collection tube) and centrifuged for 2 minutes at 13 000 rpm. After centrifugation, 300 μl Ethanol (70%) were added to the collection tube, the solution was mixed by pipetting and then transferred to an RNeasy spin column (placed in a new 2 ml collection tube) for centrifugation at 10 000 rpm for 15 s. The flow-through was discarded and the spin column placed in a new collection tube. To wash the spin column membrane 350 μl RW1 buffer were added, followed by 15 s centrifugation at 10 000 rpm. For DNase digestion, 10 μl DNase were given to 70 μl RDD buffer and placed into the spin column membrane. After 15 minutes of incubation time at room temperature, 350 μl RW1 buffer were added followed by 15 s centrifugation at 10 000 rpm. The flow was discarded and the spin column was placed in a new collection tube, followed by the addition of 500 μl RPE buffer and subsequent centrifugation for 15 s at 10 000 rpm. Discarding the flow again, the spin column was placed in a new collection tube, 500 μl RPE buffer were added and the solution was centrifuged for 2 minutes at 13 000 rpm. Finally, the spin column was placed in a 1.5 ml collection tube, 30 μl RNase free water were added followed by centrifugation for 1 minute at 10 000 rpm. The eluate contains the RNA and was frozen at −80 °C before further processing.

### Gene expression analysis

RNA quality evaluation and cDNA microarray experiments were performed according to Affymetrix standard protocols by the Genomics and Transcriptomics Lab (GTL) at the Heinrich-Heine-University Dusseldorf on Clariom S™ Assays. The generated CEL files were normalized and analyzed using the Transcriptome Analysis Console (TAC) 4.0 software. P-values were calculated using an eBayes corrected ANOVA followed by correction for multiple testing with the Benjamini-Hochberg method (FDR p-value). The thresholds for differential expression were |fold change| > 1.5 and FDR adjusted p-value < 0.05. Significantly impacted pathways were analyzed using Advaita Bio’s iPathwayGuide. This software analysis tool implements the Impact Analysis approach that takes into consideration the direction and type of all signals on a pathway, the position, role and type of every gene as described by Draghici^39^. Adjustment for multiple comparisons using the Bonferroni method was implemented during pathway analysis.

### Flow cytometry

All flow cytometry measurements were performed with a BD FACSCalibur™ flow cytometer. For evaluation, debris was removed by gating the living cells in a forward vs. sideward scatter plot (FSC vs. SSC) and for every sample at least 1 000 cellular events were recorded.

### Assessment of CD34^+^ purity and GQD uptake

Cells with and without GQDs were washed twice with PBS (2 ml, 5 min centrifugation at 300 g). In order to assess the amount of CD34^+^ cells, 10^5^ cells of every condition were incubated with 10 μl CD45-FITC/CD34-PE antibody for 15 minutes at room temperature in the dark. Cells were washed with PBS again and fixed with 200 μl 0.5% formaldehyde solution before flow cytometry analysis. CD34^+^ cells were determined out of a CD45 vs CD34 dot plot. To study the uptake of GQDs into the cells, 10^5^ of the washed cells with and without GQDs were fixed with 200 μl 0.5% formaldehyde solution. The fluorescence was measured in the FITC (488 nm exitation, 530 ± 30 nm emission) as well as in the PE (488 nm excitation, 575 ± 26 nm emission) channel, as both channels are in the emission range of the GQDs.

### Annexin V apoptosis assay

Cells were washed twice with cold PBS and incubated with 2.5 μl CD34-APC antibodies for 15 minutes at room temperature in the dark followed by one washing step. Then they were resuspended in 1x Binding Buffer at a concentration of 1 × 10^6^ cells/ml before adding 2.5 μl of FITC Annexin V and PI each per 10^5^ cells. After vortexing, the cells were incubated for 15 min at room temperature in the dark. Finally, 400 μl 1x Binding Buffer were added and the samples were measured by flow cytometry immediately.

### Measurement of intracellular Selenoprotein W

For permeabilization of the cells, the Fix and Perm Kit was used. Cells were washed twice with cold PBS and resuspended in 100 μl fixation reagent. After vortexing, the cells were incubated for 15 min at room temperature in the dark before they were washed with PBS again, followed by incubation with 100 μl permeabilization reagent and 1 μl AF647 conjugated Selenoprotein W antibody for 20 minutes at room temperature in the dark. Finally, the cells were washed with PBS, resuspended in 200 μl 0.5% formaldehyde solution and analysed by flow cytometry.

### Ethical statement

All experiments were performed in compliance with the relevant laws and institutional guidelines and have been approved by the ethical committee of the Heinrich-Heine-University Düsseldorf (study number 2018-50-FmB). All donors have given their informed consent according to the guidelines of the ethical committee specified above.

## Supplementary information

Supporting information for: The Low Toxicity of Graphene Quantum Dots is Reflected by Marginal Gene Expression Changes of Primary Human Hematopoietic Stem Cells

## Data Availability

The datasets generated during and/or analysed during the current study are available from the corresponding author on reasonable request.
