# Supplementary material for: The Low Toxicity of Graphene Quantum Dots is Reflected by Marginal Gene Expression Changes of Primary Human Hematopoietic Stem Cells
Source: Sci Rep. 2019 Aug 19;9:12028. doi: 10.1038/s41598-019-48567-6 (PMC6700176; doi:10.1038/s41598-019-48567-6)
Supplement: Supplementary file 1 — Supporting information for: The Low Toxicity of Graphene Quantum Dots is Reflected by Marginal Gene Expression Changes of Primary Human Hematopoietic Stem Cells [file 41598_2019_48567_MOESM1_ESM.pdf]

Supporting information for: The Low Toxicity of Graphene Quantum Dots is Reflected by Marginal Gene Expression Changes of Primary Human Hematopoietic Stem Cells

S. Fasbender,[†] L. Zimmermann,[†] R.-P. Cadeddu,[‡] M. Luysberg,[¶] B. Moll,[§] C. Janiak,[§] T. Heinzel,[*,†] and R. Haas[*,‡]

†*Condensed Matter Physics Laboratory, Heinrich-Heine-University, D-40204 Düsseldorf, Germany*

‡*Department of Haematology, Oncology and Clinical Immunology, Heinrich-Heine-University, D-40204 Düsseldorf, Germany*

¶*Ernst Ruska-Centre, Jülich Research Centre, 52425 Jülich, Germany*

§*Institute for Inorganic Chemistry and Structural Chemistry, Heinrich-Heine-University, D-40204 Düsseldorf, Germany*

E-mail: thomas.heinzel@hhu.de; Haas@med.uni-duesseldorf.de

The supporting information provides further data regarding the characterization of the graphene quantum dots, the cell experiments and the gene expression analysis.

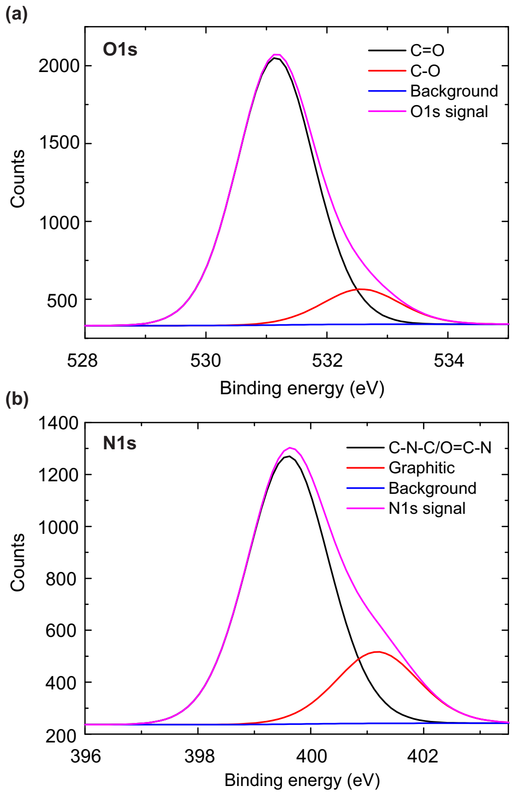

Figure S1: Fine structures of the XPS measurements at our GQDs corresponding to the (a) O1s resonance and (b) N1s resonance.

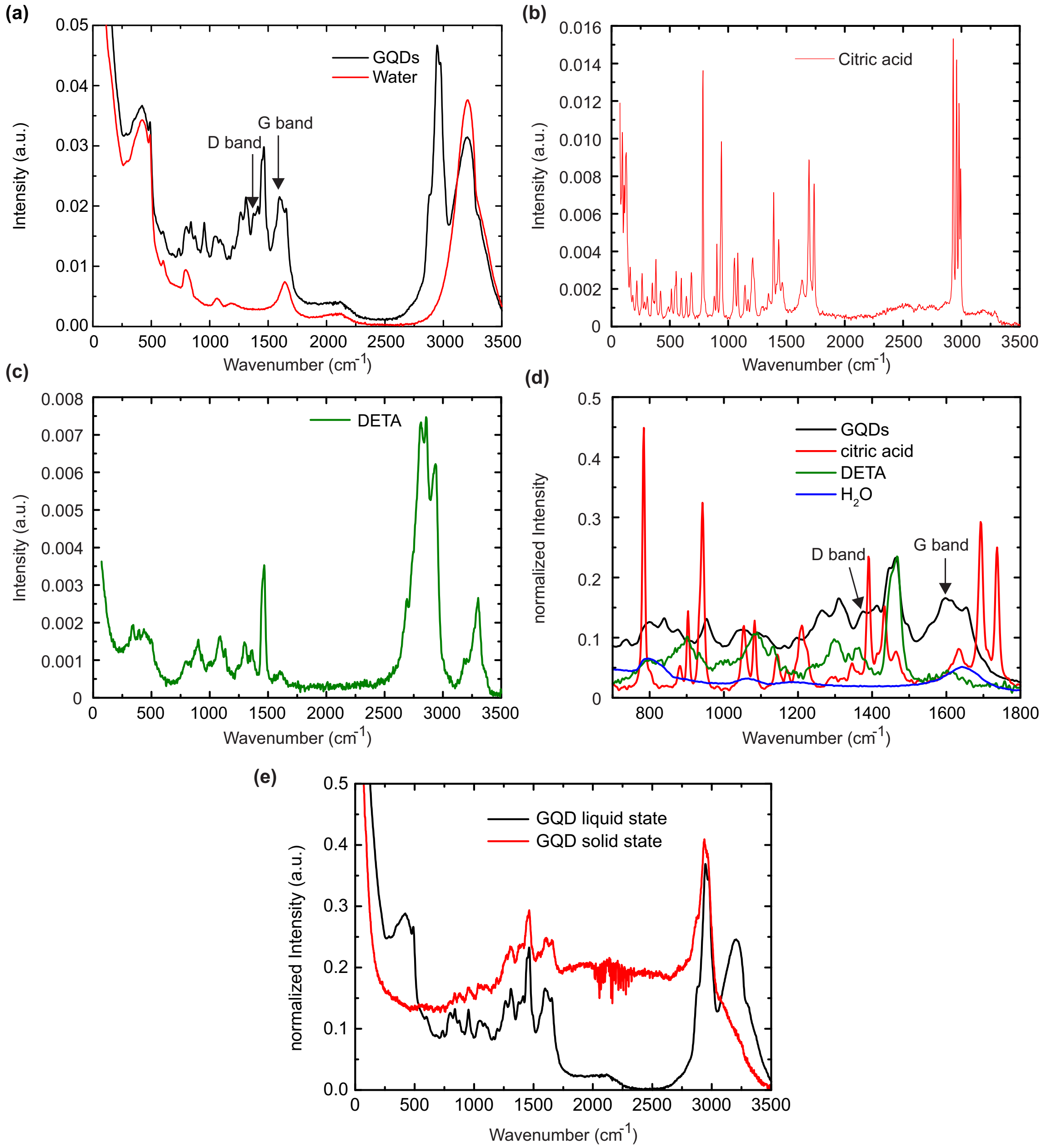

Figure S2: Raman spectra of (a) GQDs and water, (b) citric acid, (c) diethylentriamine (DETA), (d) a comparison of the spectra of GQDs, citric acid, DETA and water in the selected, relevant region from 700-1800 cm^{-1} and (e) a comparison of the spectra of dried GQDs and water-dispersed GQDs. Raman signals of the GQDs at 1657, 1411, 1053, 943 and 785 cm^{-1} match with vibrations of citric acid. Signals at 1463, 1309 and 1091 cm^{-1} match with diethylenetriamine vibrations. We can also assign signals at 2946 and 2976 cm^{-1} to vibrations of citric acid and DETA. Two small bands are visible at 1375 and 1596 cm^{-1} which we assign to the D and G band signals known for graphene quantum dots.

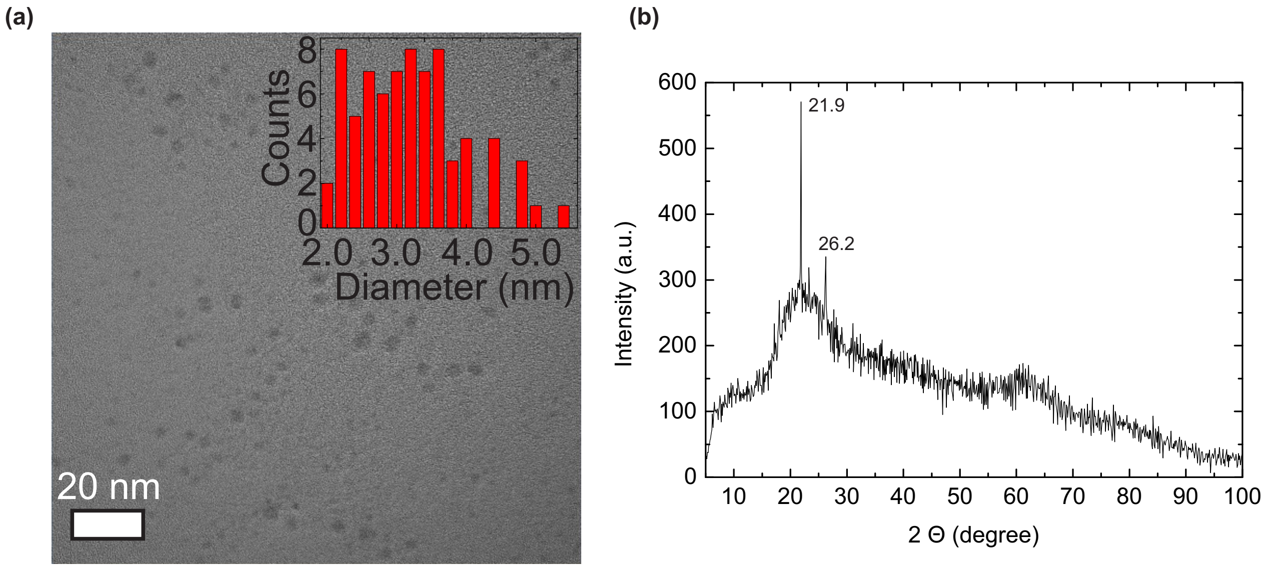

Figure S3: (a) TEM image of the GQDs and the corresponding size histogram (inset) and (b) XRD pattern of the GQDs.

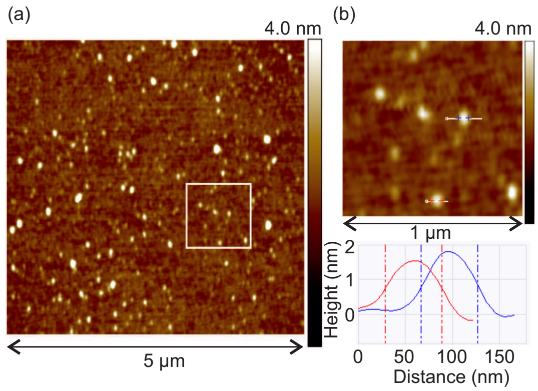

Figure S4: The GQDs deposited on SiO_2 as seen by an atomic force microscope on a larger scale (a) and a zoom with the height profiles of two GQDs (b) The height between 1 nm and 2 nm indicates up to three graphene layers.

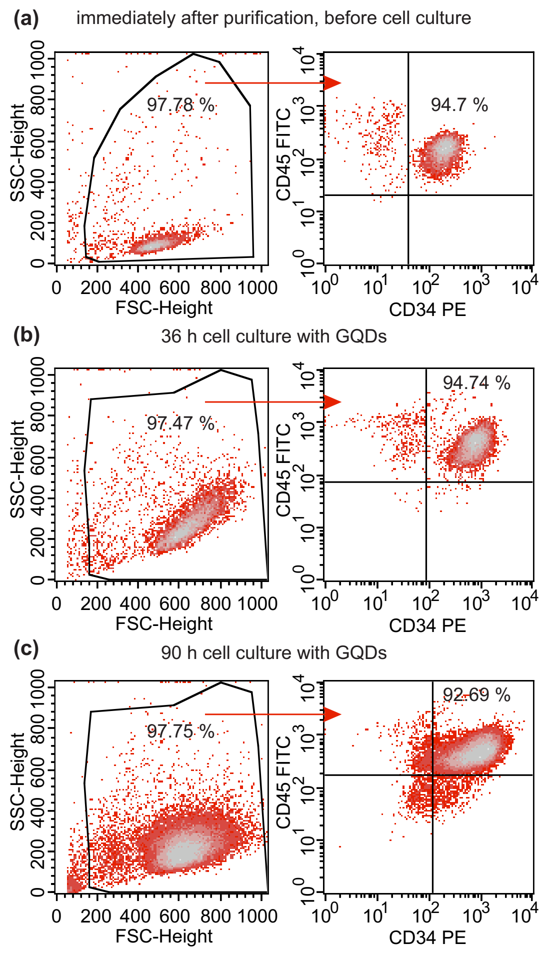

Figure S5: Fraction of $CD34^+$ following selection using anti-CD34 MoAb coated immunomagnetic beads at time point 0 (a), after 36 h (b) and after 90 h of cell culture with GQDs (c).Viable cells were gated using a forward scatter (FSC) vs side scatter (SSC) plot. The fraction of $CD34^+$ cells was obtained in a CD34 vs CD45 plot based on all viable cells.

Table S1: RIN numbers and RNA concentraion

Donor	Condition	RNA concentration (ng/μl)	RIN number
1	before cell culture	3.4	9.7
	36 h cell culture	11.5	10.0
	36 h cell culture with GQDs	8.7	10.0
2	before cell culture	7.7	8.8
	36 h cell culture	19.6	10.0
	36 h cell culture with GQDs	9.1	9.9
3	before cell culture	6.8	8.4
	36 h cell culture	17.7	10.0
	36 h cell culture with GQDs	14.9	10.0
4	before cell culture	3.4	9.7
	36 h cell culture	15.6	10.0
	36 h cell culture with GQDs	11.1	10.0

Table S2: Signaling pathways belonging to Figure 4

**Pathway name**	**Bonferroni p-value (36 h with GQDs vs. before cell culture)**	**Bonferroni p-value (36 h without GQDs vs. before cell culture)**
DNA replication	6.83E-13	9.81E-14
Metabolic pathways	1.04E-11	9.42E-13
Ribosome biogenesis in eukaryotes	2.49E-07	3.56E-07
Oxidative phosphorylation	2.32E-05	6.10E-06
Cell cycle	3.52E-05	6.74E-05
Proteasome	3.56E-05	2.43E-05
Fanconi anemia pathway	1.42E-04	1.24E-04
Epstein-Barr virus infection	3.54E-04	4.60E-04
Parkinson's disease	3.94E-04	2.26E-04
Alzheimer's disease	4.81E-04	5.43E-04
Spliceosome	5.77E-04	3.85E-03
Aminoacyl-tRNA biosynthesis	8.03E-04	5.81E-04
Mismatch repair	1.57E-03	4.30E-04
Huntington's disease	1.60E-03	1.55E-03
MicroRNAs in cancer	1.60E-03	2.14E-05
Apoptosis	1.92E-03	7.17E-02
Non-alcoholic fatty liver disease (NAFLD)	1.93E-03	2.01E-02
Nucleotide excision repair	3.00E-03	2.35E-03
Pyrimidine metabolism	3.26E-03	8.65E-04
Homologous recombination	3.59E-03	3.56E-03
RNA transport	3.76E-03	2.67E-03
Hepatitis B	5.72E-03	9.24E-01
NOD-like receptor signaling pathway	6.10E-03	5.12E-02
Citrate cycle (TCA cycle)	1.11E-02	2.47E-02
Cellular senescence	1.72E-02	2.39E-02
Viral carcinogenesis	2.76E-02	4.37E-03
Arginine and proline metabolism	3.67E-02	7.04E-01
Colorectal cancer	4.78E-02	1.00E+00
Purine metabolism	4.80E-02	6.97E-04
Protein processing in endoplasmic reticulum	7.48E-02	3.45E-02
Biosynthesis of amino acids	1.96E-01	3.94E-02
HTLV-I infection	3.14E-01	4.97E-02

Table S3: Gene expression changes belonging to Figure 6

Gene symbol	Fold change	p-value	FDR p-value	Description
SEPW1	-5.14	1.64E-08	4.00E-04	selenoprotein W, 1
FRMD3	-2	9.46E-05	0.9998	FERM domain containing 3
TYRO3	-1.92	0.0357	0.9998	TYRO3 protein tyrosine kinase
LRRCC1	-1.73	0.003	0.9998	Entrez Gene ID(s) 85444
CELF4	-1.68	0.0031	0.9998	CUGBP, Elav-like family member 4
CD53	-1.63	0.0472	0.9998	Memczak2013 ALT_ACCEPTOR,
GNG3	-1.63	0.0256	0.9998	guanine nucleotide binding protein (G protein), gamma 3
KIF2C	-1.6	0.0258	0.9998	kinesin family member 2C
LINC01140	-1.57	0.0229	0.9998	long intergenic non-protein coding RNA 1140
PLEKHA8	-1.56	0.002	0.9998	pleckstrin homology domain containing, family A member 8
ZNF780A	-1.56	0.0187	0.9998	zinc finger protein 780A
NUDT2	-1.54	0.0383	0.9998	nudix hydrolase 2
GAS1	-1.54	0.0048	0.9998	growth arrest-specific 1
KRTAP21-1	-1.53	0.0289	0.9998	keratin associated protein 21-1
AC018462.2;	1.5	0.0035	0.9998	Salzman2013 ANTISENSE, coding
GPR135	1.52	0.0152	0.9998	G protein-coupled receptor 135
ZNF345	1.52	0.0435	0.9998	zinc finger protein 345
HMBS	1.53	0.0422	0.9998	hydroxymethylbilane synthase
ZBED8	1.53	0.0356	0.9998	zinc finger, BED-type containing 8
NBEAL1	1.54	0.0132	0.9998	neurobeachin like 1
LOC100133315; RP11-849H4.2	1.54	0.0222	0.9998	transient receptor potential cation channel subfamily C, member 2-like
ZNF582	1.58	0.0378	0.9998	zinc finger protein 582
TNXA	1.59	0.004	0.9998	tenascin XA (pseudogene)
ARAP2	1.59	0.0115	0.9998	ArfGAP with RhoGAP domain
SMC1B	1.59	0.0059	0.9998	structural maintenance of chromosomes 1B
BBS5	1.61	0.0215	0.9998	Bardet-Biedl syndrome 5
ASIC2	1.61	6.00E-04	0.9998	acid sensing ion channel 2
SLC26A11	1.62	0.0246	0.9998	solute carrier family 26 (anion exchanger)
RAI14	1.63	0.0401	0.9998	Entrez Gene ID(s) 26064
KRTAP2-4	1.67	0.012	0.9998	keratin associated protein 2-4
DGKD	1.68	0.0035	0.9998	diacylglycerol kinase, delta 130kDa
BBOF1	1.76	0.0059	0.9998	basal body orientation factor 1
ST8SIA4	1.8	0.0422	0.9998	ST8 alpha-N-acetyl-neuraminide alpha-2,8-sialyltransferase 4
